# The evolution of birdsong on islands

**DOI:** 10.1002/ece3.864

**Published:** 2013-11-22

**Authors:** Jennifer Morinay, Gonçalo C Cardoso, Claire Doutrelant, Rita Covas

**Affiliations:** 1 AgroParisTech16 Rue Claude Bernard, 75005, Paris, France; 2 CEFE-CNRS1919 Route de Mende, 34293, Montpellier Cedex 5, France; 3 CIBIO, Research Centre in Biodiversity and Genetic Resources, Campus Agrário de Vairão, Rua Padre Armando Quintas4485-661, Vairão, Portugal; 4 Biology Department, Science Faculty, University of PortoPorto, Portugal

**Keywords:** Insularity syndrome, ornaments, sexual selection, sexual signals, species recognition

## Abstract

Islands are simplified, isolated ecosystems, providing an ideal set-up to study evolution. Among several traits that are expected to change on islands, an interesting but poorly understood example concerns signals used in animal communication. Islands are typified by reduced species diversity, increased population density, and reduced mate competition, all of which could affect communication signals. We used birdsong to investigate whether there are systematic changes in communication signals on islands, by undertaking a broad comparison based on pairs of closely related island-mainland species across the globe. We studied song traits related to complexity (number of different syllables, frequency bandwidth), to vocal performance (syllable delivery rate, song duration), and also three particular song elements (rattles, buzzes, and trills) generally implicated in aggressive communication. We also investigated whether song complexity was related to the number of similar sympatric species. We found that island species were less likely to produce broadband and likely aggressive song elements (rattles and buzzes). By contrast, various aspects of song complexity and performance did not differ between island and mainland species. Species with fewer same-family sympatric species used wider frequency bandwidths, as predicted by the character release hypothesis, both on continents and on islands. Our study supports the hypothesis of a reduction in aggressive behavior on islands and suggests that discrimination against closely related species is an important factor influencing birdsong evolution.

## Introduction

Islands are isolated and simplified ecosystems, with reduced number of habitats and species, and hence provide unique opportunities to study evolutionary patterns and processes (MacArthur and Wilson 1967; Losos and Ricklefs 2009). The basic ecological features of islands lead to a set of convergent demographic and evolutionary changes often referred to as “insularity syndrome”. Specifically, decreased species diversity and interspecific competition on islands leads to broader ecological niches of species and to “density compensation”, whereby island populations live at higher densities than on the mainland (MacArthur et al. 1972; Blondel et al. 1988). In addition, adaptation to insular environments usually leads to the evolution of morphological (Lomolino 2005; Price and Philimore 2007; Fleischer and James 2008) and life-history adaptations, such as reduced metabolic rate (McNab 1994; McNab and Ellis 2006), reduced fecundity and extended parental care (MacArthur and Wilson 1967; Covas 2012), higher survival (Adler and Levins 1994; Whittaker and Fernandez-Palacios 2007), increased sociality (Covas 2012), decreased sexual selection (Griffith 2000), and reduced territoriality (Stamps and Buechner 1985).

These peculiarities of island ecosystems and life histories may set the stage for the evolution of yet other traits, in particular sexual and social signals used in communication, but this has seldom be quantified. A particularly interesting but poorly understood case is the evolution of birdsong on islands. Avian songs are important for individual and species recognition (Seddon 2005) and play an important role in sexual and social communication (Catchpole and Slater 1995). Birdsong evolution is also strongly constrained and affected by morphology and habitat type (Seddon 2005; Boncoraglio and Saino 2007). On islands, birdsong may differ for several reasons. First, islands are species poor compared with mainland areas. One of the functions of signals is to code for species identity, and it has been shown that the variability of signals is influenced by the similarity and number of closely related species sharing the same habitat (e.g., Miller 1982; Kroodsma 1985; Naugler and Ratcliffe 1994; Doutrelant et al. 2000; Seddon 2005). Living in habitats with lower number of species, particularly closely related species, makes the task of species recognition easier and should lead to changes in signals diversity. In the case of birdsong, it could lead to increased acoustic diversity (character release hypothesis; Kroodsma 1985; Naugler and Ratcliffe 1994).

Second, the increased population density that typifies most islands could affect signal evolution. Island vertebrates living at high population densities often show reduced aggression toward conspecifics (Stamps and Buechner 1985). This could arise through nonexclusive mechanisms such as increased resource abundance (due to lower species diversity on islands) or elevated costs of aggression or territory defense when encounters with conspecifics are very frequent due to higher densities (reviewed in Stamps and Buechner 1985).

Finally, mate choice (i.e., intersexual selection) also influences the evolution of birdsong and is expected to be consistently lower on islands. The lower genetic diversity of island populations (Frankham 1997) should decrease the genetic benefits of mate choice, thus reducing the strength of intersexual selection (Brown 1997; Petrie et al. 1998). This is supported by lower rates of extra-pair paternity on islands (Griffith 2000). In addition, life-history shifts toward greater investment in parental care on islands, including male care (Covas 2012) and increased survival (Adler and Levins 1994; Whittaker and Fernandez-Palacios 2007), may lead to reduced ornamentation due to trade-offs between investment in parental care or survival versus in costly sexual ornaments (Scott and Clutton-Brock 1990; Figuerola and Green 2000; Dunn et al. 2001; Magrath and Komdeur 2003). To date, predictions of decreased secondary sexual traits on islands have been supported by studies of plumage dichromatism (Fitzpatrick 1998; Figuerola and Green 2000).

Hence, the island environment could have different effects on the evolution of birdsong. Previous work comparing birdsong on islands versus continents, mostly comparisons of repertoire sizes, has not yet revealed general patterns (reviewed in Price 2008). Almost all such studies have looked at differences within a single species (only one, of 15 studies reviewed by Price 2008; compared two closely related species; Mirsky 1976). Short-term phenomena that may affect these within-species comparisons, such as cultural bottlenecks (Thielcke 1973) or withdrawal from song learning (Baker et al. 2006) in species that learn song socially, may not translate into longer term differences among species, and a robust cross-species test of insularity syndrome in birdsong has not yet been performed.

Here, we conducted paired comparisons of insular passerines from around the world with closely related mainland species to determine whether there are general patterns of song evolution on islands. We investigated whether specific song traits differ between island and mainland species and, in addition, tested whether living in sympatry with closely related species (e.g., Naugler and Ratcliffe 1994; Seddon 2005) can explain part of these differences. Vocal evolution can also be affected by acoustic properties of the habitats (Morton 1975; Wiley and Richards 1982; Wiley 1991; Slabbekoorn and Smith 2002; Naguib 2003; Boncoraglio and Saino 2007), body size (Wallschläger 1980; Ryan and Brenowitz 1985), and latitude (Irwin 2000; Cardoso et al. 2012; Weir et al. 2012), and hence, we also analyzed and controlled for these effects.

Specifically, we investigated song traits related to complexity (number of different syllables, frequency bandwidth) and vocal performance (syllable delivery rate, song duration), as both song complexity and performance can be used in mate choice and territory defense (Catchpole and Slater 1995; Gil and Gahr 2002) and in species recognition. We also investigated the presence of three particular song elements—rattles, buzzes, and trills—known to have salient roles in aggressive male–male interactions (e.g., Smith 1959; Morton 1977; Rehsteiner et al. 1998; Trillo and Vehrencamp 2005; Benedict et al. 2012). Trills, or aspects of trill performance, can in addition be preferred by females (Vallet and Kreutzer 1995; Vallet et al. 1998; Drăgănoiu et al. 2002; Ballentine et al. 2004).

Our predictions were the following. To the extent that song complexity, performance, or the presence of aggressive elements is linked to mate choice and territoriality, the hypothesis of decreased territoriality and relaxed mate choice on islands predicts a decrease in those song traits compared with mainland species. Song also codes for species recognition and thus, to the extent that species recognition is eased on islands under the character release hypothesis (Kroodsma 1985; Naugler and Ratcliffe 1994), we expect the opposite pattern of increased song complexity on islands. This latter hypothesis also predicts that differences in song complexity are explained by the number of closely related species living in sympatry.

## Materials and Methods

### Species pairs and song data

The study was based on pairs of island and mainland passerines, following the same method as Covas (2012). In brief, we identified pairs made up of an island endemic species and its most closely related continental species for which there were also data available (Appendix A1), chosen based on molecular phylogenies or on taxonomy as a proxy for relatedness. If several continental species were good candidates, we matched the pairs by latitude and geographic proximity. In two cases, this resulted that the same continental species was paired to two island species, but those were cases of independent colonizations rather than colonization followed by an insular radiation, and therefore should reflect independent evolutionary events. We only included islands smaller than 12,000 km² to avoid pseudo-continental ecosystems (Blondel 2000; Lomolino 2005) and, given its size, Papua New Guinea was used as a continental area in comparison with nearby islands.

We used 49 pairs of passerine species for which we could obtain good quality (i.e., measurable) song recordings from the Macaulay Library (Cornell Lab of Ornithology, http://macaulaylibrary.org) or the Xeno Canto online database (http://www.xeno-canto.org). We selected up to 5 recordings per species (on average 3.02 ± 1.49 SD) based on sound quality and geographical location (on the same island or the same continental region) and, depending on the length and quality of recordings, analyzed up to 10 songs per recording (on average 6.40 ± 3.46 SD). When there were less than five good quality recordings from the Macaulay Library, we complemented the search with recordings from the Xeno Canto online database. We selected recordings of songs rather than other types of vocalizations (e.g., calls, mechanical sounds) and did not use recordings of juveniles, which might still be in their song learning phase. When necessary, we consulted written descriptions of songs (Del Hoyo et al. 2004; BirdLife International 2012) to distinguish song from other types of vocalizations. Individual songs were identified as a group of syllables separated from other songs by at least three times the typical intersyllable intervals in the recording.

**Figure 1:**
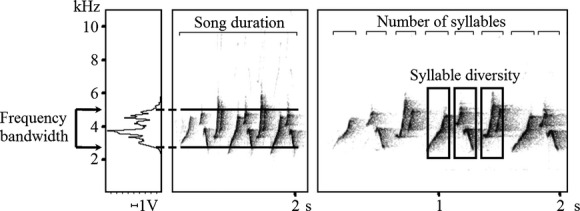
Spectrograms from a song of *Geothlypis rostrata*, illustrating song measurements. Frequency bandwidth was calculated from measurements of maximum and minimum song frequency (frequencies at which the sound amplitude drops 17 dB below the song peak amplitude) on the power spectrum of song (left panel: amplitude in volts, by frequency in kilohertz). Song duration was measured on spectrograms (middle panel: time in seconds, by frequency in kilohertz), and total number of syllables (to calculate syllable rate) and number of different syllable per song were counted on spectrograms with higher time resolution (right panel; in this example, 8 syllables and 3 different syllables).

Recordings were downsampled to 22.05 kHz, high-pass filtered using thresholds below song minimum frequency and analyzed on Avisoft SASLab Pro v.5.1.23 (Avisoft Bioacoustics, Berlin, Germany). We used power spectra for frequency measurements, and spectrograms with a FFT length of 512 Hz and Hamming window with 50% overlap (corresponding to 11.6 ms by 43 Hz resolution, Fig. 1) for the remaining measurements.

We measured 4 song parameters (Fig. 1): *song duration*, *frequency bandwidth*, *syllable rate*, and number of *different syllables per song*. *Song duration* and *syllable rate* are closely related to aspects of vocal performance (singing longer songs or with fast syllable rate); *frequency bandwidth* and number of *different syllables per song* are related to song complexity (diversity of sounds used within songs). To obtain these measurements, we marked individual songs on spectrograms and then used automatic measurement tools. We obtained *song duration*, in seconds, from these markings. We calculated *frequency bandwidth* on a logarithmic scale (i.e., a ratio scale): we first obtained maximum and the minimum frequencies for each song as described below, log transformed them and calculated their difference. This provides more biologically meaningful measurements, because vertebrates perceive sound frequency on a logarithmic scale, and the relation between resonating frequency of the avian vocal tract and its size or behavioral adjustments during singing is also logarithmic (Cardoso 2013). Maximum and minimum frequencies were identified as the frequencies at which the sound amplitude drops 17 dB below the song peak amplitude (amplitude of the loudest frequency), which captures the vast majority of sound energy in songs while being generally robust to interference by background noise in our recordings. We checked the correctness of measurements visually on spectrograms and removed bursts of noise that affected frequency measurements. On spectrograms with higher time resolution (75% window overlap corresponding to 43 Hz resolution), we visually counted the number of syllables per song and then divided it by song duration to obtain *syllable rate*. We did not analyze number of syllables per song as an independent song trait because it is highly dependent on song duration (bivariate correlation using species mean = 0.89). We also visually counted the number of different syllables per song, which is a measure of *syllable diversity*. A syllable was defined as a single note or a tight group of notes clearly separated from other syllables by a visible temporal pause at the above resolution. Measurements of each song trait were averaged per recording.

**Figure 2:**
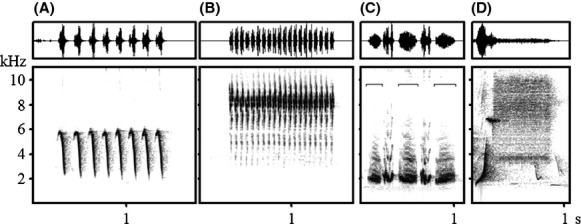
Spectrograms illustrating (A) trills (in a song of *Troglodytes beani*), (B) rattles (in a song of *Tiaris olivaceus*), and (C, D) buzzes (*Mimus gilvus*, three buzzes marked with brackets; and *Loxigilla portoricensis*, a buzz preceded by a whistle). Upper panels show the signals waveforms of these songs.

In addition, we noted whether each recording included at least one *rattle*, *buzz* or *trill* (Fig. 2). Trills, rattles, and buzzes all refer to repetitions or pulsation of sounds, differing in rate, and other phonological properties. These terms are used in somewhat different ways in the literature. Here, we define them as follows. In trills, the repeated unit is a regular syllable, of variable complexity, separated from similar syllables in the trill by time intervals of the same magnitude than the intervals between other syllables in song. Rattles repeat simple units of wide frequency range, much shorter than syllables, and often separated by equally short time intervals. Buzzes are wide frequency range sounds with amplitude pulses at typically over 100 Hz.

### Morphological and ecological data

We obtained the mean body mass for each species from Del Hoyo et al. (2004) or, when not available in the former, from Dunning (2008), using median values when only ranges were reported or when masses were reported separately for males and females. Body mass did not differ significantly between island and mainland species (pairwise comparisons using Wilcoxon rank-sum test, *P* > 0.85).

We defined three categories of vegetation density to classify the predominant breeding habitat of each species, based on their habitat descriptions in Del Hoyo et al. (2004). We assigned to *Closed* habitats the species living mostly in high vegetation (e.g., forest, woodland, forest edges, plantations, mangroves, jungle), to *Open* habitats those species living mostly in low vegetation (e.g., shrub, scrub, bush, savannas, grassland, steppes, desert), and to *Intermediate* those species described as usually living in both closed and open habitat. Habitat type did not differ between island and mainland species (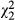 = 1.77, *P* = 0.41).

For each species, we counted the number of sympatric species in the same family, that is, those whose wintering or breeding distribution overlapped with the focal species. We used the distribution maps in Del Hoyo et al. (2004). Same-family sympatric species numbers were significantly lower on islands (2.31 ± 2.03 SD; from 1 to 11) than mainland areas (19.32 ± 18.49 SD; from 1 to 80; pairwise comparison using Wilcoxon rank-sum test, *P* < 0.001).

**Figure A1:**
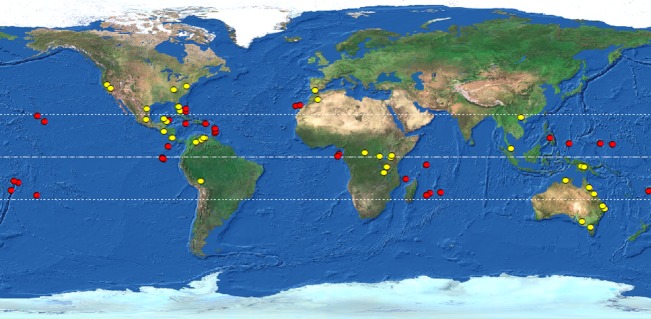
Geographical locations for the recordings of each insular (in red) and mainland species (in yellow; mainland locations are the mean for the different recordings of each species). Dashed lines mark the equator and the tropics.

We obtained the absolute value for latitude of each island (UNEP 2010) and the average latitude value of the mainland sites where the recordings were made. Absolute values of latitude were comprised between 0.3 and 28.4° (mean 14.2 ± 8.4° SD) and thus were biased toward the tropical region (see Appendix Table A1 and Appendix Fig. A1) because of the higher species richness and higher number of islands in the tropics.

### Analyses

We compared each song trait within pairs of closely related island and mainland species, which is a simple method to test for an effect of insularity with incomplete phylogenetic information (Møller and Birkhead 1992; Nunn 2011; Covas 2012). In order to include additional factors (the morphological and ecological traits mentioned above) in the comparative models, we ran the paired comparisons within a generalized linear mixed-effects model (GLMM) framework, using the R packages *lme4* and *nlme* (Bates et al. 2011; Pinheiro et al. 2011). GLMMs had a nested random effect of the form “species” nested in “pair” nested in “family”, and the statistical units were the song measurements per recording. The nested structure approximates the phylogenetic structure of the data, and using values per recording nested within species, rather than species means, accounts for within-species variation and differences in sample sizes among species (e.g., Felsenstein 2008), while keeping statistical testing at the appropriate species level. This approach also weights the analysis by the robustness of species means, and thus by sample size (number of recordings per species). Based on a graphical assessment of residuals, and to insure their normality, we log-transformed *song duration*, *syllable rate,* and *syllable diversity*. The presence or absence of trills, rattles, and buzzes was analyzed using binomial distributions. Within-species repeatability for measured song traits, with values per recording as statistical units, was generally high: 0.59 for frequency bandwidth, and over 0.75 for all others (repeatability for continuous traits calculated as in the study by Lessells and Boag 1987 and for the binomial variables, as intraclass correlations, Zuur et al. 2009).

For each response variable (song trait), we ran a model that included latitude and body mass as covariates, vegetation density as a categorical factor, and insularity as a dichotomous factor. We also included the interaction between insularity and latitude as there could be a stronger response to insularity at higher latitudes (see Covas 2012). This model did not include the variable “number of sympatric species in the same family”, because this is strongly collinear with insularity (see above). To analyze the effect of sympatric species, we ran a second model removing the factor insularity (and its interaction term) and instead adding the covariate “number of sympatric species in the same family”. All statistical analyses were conducted in R v.2.13.1 (R Development Core Team 2011). Model selection was based on F-tests, and we used backward deletion conserving marginally significant (*P* < 0.1) variables. We assessed whether type I error due to stepwise model selection could affect our conclusions (Mundry and Nunn 2009) by reporting results of both the full and final models. The full model approach is recommended for nonpredictive models, while the stepwise approach gives more reliable estimates of the effect of significant variables.

## Results

### Insularity

**Figure 3:**
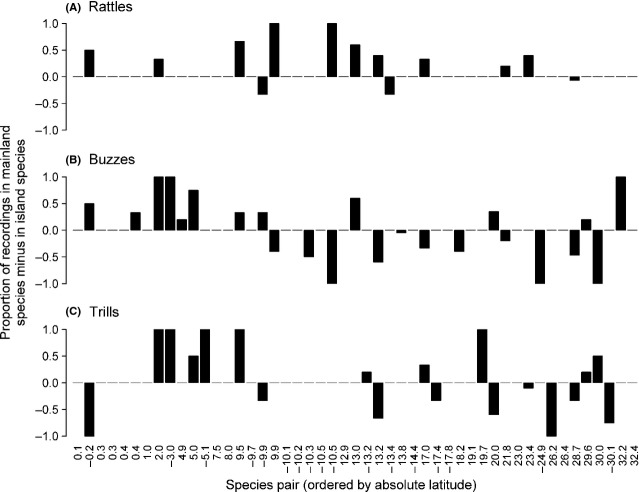
Differences in the proportion of song recordings with (A) rattles, (B) buzzes, and (C) trills in paired mainland and island species. Mean latitude for each species pair is indicated below the horizontal axis (southern latitudes as negative values). Note that species pairs are weighted differently in the comparative analysis, due to differences in sample sizes (number of recordings). To visualize sample sizes, see Appendix Fig. A2.

The occurrence of rattles and buzzes was lower in recordings of island than mainland species (rattles: 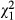 = 3.96, *P* = 0.047; buzzes: 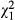 = 4.94, *P* = 0.026, *n* = 298; Table 1). In the case of rattles, this insularity effect was only significant in the final model, after the nonsignificant effects of mass and vegetation density were removed (Table 1). Nonetheless, combining rattle and buzz into a single variable, the effect of insularity is significant in the full and selected models (result not shown). The interaction between latitude and insularity was significant for buzzes and marginally significant for rattles (rattles: 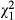 = 3.23, *P* = 0.072, Fig. 3A; buzzes: 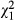 = 5.39, *P* = 0.020, Fig. 3B; Table 1), indicating that the insular effect is attenuated with increasing absolute latitude, and for buzzes may even reverse at higher latitudes (Fig. 3B). However, there are only four insular species in our dataset above 23 degrees of absolute latitude (i.e., outside of the tropics), so this result should be taken with caution.

**Table 1:** Presence of rattles, buzzes, or trills in song recordings of passerine species, relative to island living, latitude, body mass, and vegetation density of habitats. Results of full and reduced GLMMs paired by species are presented

	Rattles		Buzzes		Trills	
			
	Full model	Final model	Full model	Final model	Full model	Final model
Insularity	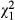 = 2.06 (0.152)	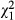 = 3.96 (**0.047**)	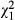 = 4.51 (**0.034**)	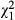 = 4.94 (**0.026**)	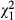 = 0.03 (0.875)	
Latitude	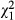 = 3.02 (0.082)	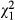 = 3.43 (0.064)	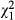 = 2.96 (0.085)	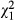 = 3.12 (0.078)	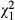 < 0.01 (0.953)	
Insularity × latitude	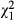 = 1.70 (0.192)	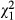 = 3.23 (0.072)	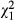 = 5.36 (**0.021**)	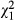 = 5.39 (**0.020**)	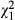 = 0.02 (0.893)	
Mass	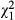 = 1.07 (0.302)		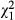 < 0.01 (0.987)		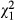 = 0.41 (0.520)	
Vegetation density	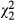 = 2.96 (0.227)		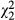 = 0.75 (0.686)		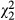 = 6.23 (**0.044**)	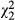 = 8.73 (**0.013**)

Indicated are χ² statistics for each factor or covariate and *P*-values (significant values in bold).

There were no significant differences between island and mainland species in the occurrence of trills (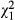 = 0.03, *P* = 0.875; Fig. 3C; Table 1). We also found no consistent differences between island and mainland species in any of the measurements of song complexity or performance (i.e., song duration, frequency bandwidth, syllable rate, and syllable diversity, Table 2).

**Table 2:** Aspects of song complexity and performance relative to island living, latitude, body mass, and vegetation density of habitats. Results of full and reduced GLMMs paired by species are presented

	Song duration		Frequency bandwidth*	Syllable rate		Syllable diversity*
				
	Full model	Final model	Full model	Full model	Final model	Full model
Insularity	*F*_1,37_ = 0.03 (0.865)		*F*_1,37_ = 3.25 (0.079)	*F*_1,37_ = 0.68 (0.414)		*F*_1,37_ = 1.06 (0.310)
Latitude	*F*_1,37_ = 0.13 (0.724)		*F*_1,37_ = 1.28 (0.264)	*F*_1,37_ = 0.52 (0.477)		*F*_1,37_ = 0.23 (0.634)
Insularity x latitude	*F*_1,37_ = 0.23 (0.637)		*F*_1,37_ = 1.24 (0.272)	*F*_1,37_ =0.72 (0.402)		*F*_1,37_ = 0.40 (0.530)
Mass	*F*_1,37_ = 10.6 (**0.002**)	*F*_1,42_ = 13.5 (**<0.001**)	*F*_1,37_ = 2.18 (0.149)	*F*_1,37_ = 10.8 (**0.002**)	*F*_1,42_ = 12.3 (**0.001**)	*F*_1,37_ = 0.02 (0.884)
Vegetation density	*F*_2,37_ = 1.57 (0.221)		*F*_2,37_ = 0.47 (0.627)	*F*_2,37_ = 1.44 (0.251)		*F*_2,37_ = 0.04 (0.965)

Indicated are *F* statistics for each factor or covariate and *P* values (significant values in bold).

* No significant factors or covariates after model selection for frequency bandwidth and syllable diversity.

### Effect of morphology and habitat

Body mass was negatively related to syllable rate and positively related to song duration (syllable rate: *F*_1,42_ = 12.3, *P* = 0.001; song duration: *F*_1,42_ = 13.5, *P* < 0.001; Table 2). Body mass had no significant effect on the other song traits (Tables 1 and 2).

Vegetation density had a significant effect on the production of trills (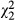 = 8.73, *P* = 0.013; Table 1), with trills less frequent in species of closed and intermediate habitats (respectively, 27 and 18%) than open habitats (49%). We found no significant effect of the vegetation density index on the other song traits (Tables 1 and 2).

### Effect of sympatric species

**Figure 4:**
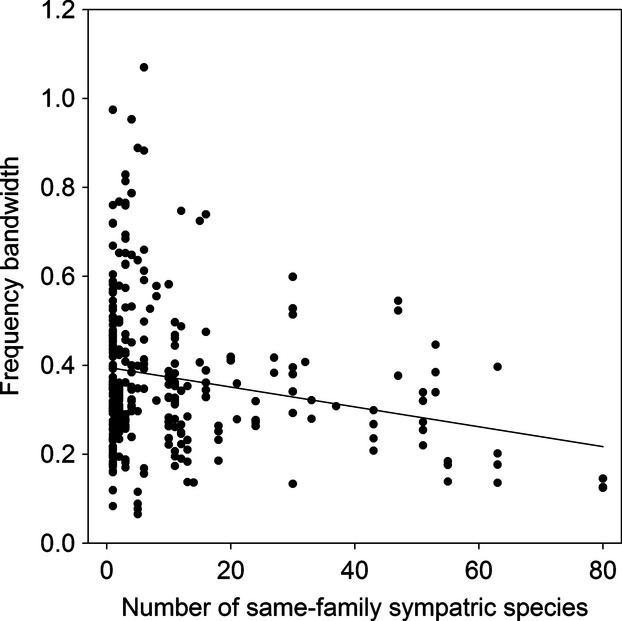
Plot of the frequency bandwidth of recordings against the number of same-family sympatric species.

With statistical models including number of sympatric species from the same family (instead of insularity), the number of sympatric species was negatively related to frequency bandwidth of songs (*F*_1,48_ = 7.85, *P* = 0.008; Fig. 4, Appendix Table A2). Number of sympatric same-family species had no significant effect on the other variables tested. The effects of the other factors or covariates were qualitatively identical to the models in the previous sections except for rattles, whose trend to increase with latitude (Table 1) was now significant (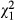 = 6.94, *P* = 0.009; Appendix Table A2), and which was now related to the vegetation density of habitats (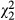 = 11.2, *P* = 0.004; Appendix Table A2), with rattles more common in open habitats (51% of recordings with rattles, compared with 12% and 11% in intermediate and closed habitats).

## Discussion

We found that island species are less likely to have rattles and buzzes in their songs than their close mainland relatives. On the other hand, trills and various aspects of song complexity and performance did not differ consistently between island and mainland species. In addition, passerines inhabiting areas with fewer same-family sympatric species used a wider frequency bandwidth, indicating higher spectral diversity within their songs, as predicted by the character release hypothesis. Finally, as expected, differences among species in body size and vegetation density of habitats influenced song duration, syllable rate, and production of trills.

Rattles and buzzes are nontonal, broadband sounds, whose acoustic properties match theoretical expectations for aggressive signals (Morton 1977), and several bird species have been shown to use them in aggressive contexts (Smith 1959; Morton 1977; Galeotti et al. 1997; Rehsteiner et al. 1998; Trillo and Vehrencamp 2005; Benedict et al. 2012). Increased aggressiveness in birds is often related to increased male–male competition for territory, mates, or food (Andersson 1994). Accordingly, polygynous species with lekking systems, where competition for mates is most intense, also use this type of nontonal sounds within their songs or courtship vocalizations more than monogamous species (Loffredo and Borgia 1986). Therefore, the lower frequency of rattles and buzzes in island passerines is consistent with the hypothesis of reduced aggression toward conspecifics and, possibly, reduced territoriality on islands. In particular, island species have been suggested to be less territorial and more tolerant of subordinates. This may arise because of increased resource abundance (as a result of lower species diversity), which would decrease the pay-off of territory defense, and because of higher intraspecific densities, which would increase the frequency of encounters with conspecifics and hence the costs of sustained intolerance toward conspecifics (reviewed in Stamps and Buechner 1985).

The island-mainland differences in the occurrence of rattles and buzzes appeared to diminish with increasing absolute latitude. This is unexpected because the ecology of islands and continents is most different at higher latitudes. In particular, the lower seasonality of islands when compared to the mainland is more evident at higher latitudes, and the effects of insularity on life-history traits such as fecundity are also more marked at higher latitudes (Covas 2012). In addition, endemic island passerines are almost always nonmigratory, which contrasts with the abundance of migratory species at higher latitudes in continents. Migration and seasonality have been related to increased elaboration in avian ornaments and songs (Bailey 1978; Fitzpatrick 1994; Spottiswoode and Møller 2004; Botero et al. 2009), possibly due to their effects on breeding synchrony and the opportunity for extra-pair mating (Thusius et al. 2001; Albrecht et al. 2007; Stewart et al. 2010). Therefore, we would expect that island–continent differences in song would be more pronounced, rather than attenuated, at higher absolute latitudes. Our finding in the opposite direction should be taken with caution, because at the global scale, the majority of islands with endemic species are tropical (and our dataset contained only 5 island species outside the tropics), and also because there may be correlates of latitude not accounted for here. For example, colonization of islands might be more frequent at higher latitudes, where migratory movements are larger, and island–continent species pairs thus might be younger and with less time for their song to diverge (more generally, sister species tend to be younger at higher absolute latitudes; Weir and Schluter 2007).

Similarly to rattles and buzzes, trills have been related to aggressive behavior (e.g., Leitão and Riebel 2003; Leitão et al. 2006; DuBois et al. 2009; de Kort et al. 2009), and, in some species, aspects of trill performance such as syllable rate or frequency bandwidth are preferred by females in mate choice tests (e.g., Vallet et al. 1998; reviewed in Podos et al. 2004). Therefore, the hypothesis that sexual selection diminishes in islands would also predict fewer trills on island species, which we did not find. This may be because, unlike for rattles and buzzes which can be short in length, trills reduce song complexity: for songs of the same length, the more trills (repetitions of the same syllable), the fewer different syllables in the song. This might prevent systematic differences in trills between island and mainland counterparts as higher sexual selection in continents could in some species select for more trills, while in others, it could select for syllable diversity and thus reduce trills (Cardoso and Hu 2011).

Complex songs with diverse syllables are also thought to be a product of mate choice (Catchpole and Slater 1995). But song complexity and syllable rate (an aspect of song performance) did not differ between island and mainland species. This contrasts with other sexual traits that were on average reduced on islands, such as sexual dichromatism (Fitzpatrick 1998; Figuerola and Green 2000; Griffith 2000). Similarly, to our more systematic comparison, previous studies that compared song of island and mainland populations have also not found consistent differences in song complexity (reviewed in Price 2008). This could either indicate that the strength of mate choice is not lower on islands or that song complexity is not a good proxy of the strength of mate choice. The latter interpretation is likely because, while indexes of sexual selection are positively related to song complexity in some groups of closely related species (e.g., Mountjoy and Leger 2001; Botero et al. 2009; Cardoso et al. 2012), in passerines at large, the intensity of sexual selection fails to explain differences in song complexity (Garamszegi and Møller 2004), and in many species, female choice does not select for more complex songs (Byers and Kroodsma 2009; Soma and Garamszegi 2011). Instead, mate choice can select for a variety of different acoustic traits (Gil and Gahr 2002), and trade-offs between song complexity and some of those acoustic traits are known (e.g., trade-off with sound amplitude, Cardoso and Mota 2009; Cardoso 2010; or with trill performance, Cardoso and Hu 2011), which may prevent predictable evolution of song complexity with increasing sexual selection.

Founder effects would predict reduced song complexity on islands for passerines that learn songs socially (Thielcke 1973). This is because when few individuals first colonize islands, they only carry a small subset of their populations' song diversity, which is then transmitted culturally to the following generations. Founder effects on song diversity can be relatively long lasting (e.g., Parker et al. 2012; reviewed in Price 2008) but, counteracting them, song complexity can build up again across a few generations of song learning (e.g., Gardner et al. 2005; Fehér et al. 2009). Therefore, it is not surprising that at our level of analyses—species differences—no reduced song complexity is detected on islands.

We found that the number of same-family sympatric species negatively influenced the frequency bandwidth of songs. This is consistent with the character displacement and character release hypotheses. High diversity of closely related species in sympatry can lead to character displacement, whereby acoustic space is partitioned, to facilitate species recognition (Doutrelant et al. 2000; Seddon 2005; Luther 2009; but see Cardoso and Price 2010). Conversely, lower numbers of closely related sympatric species allow character release, whereby songs are more variable (Kroodsma 1985; Naugler and Ratcliffe 1994). Despite on average islands having fewer same-family species than mainland, frequency bandwidth did not differ significantly between islands and continents. This indicates that character release in frequency bandwidth of song is not a phenomenon particularly strong on islands compared with continents.

Finally, body mass and the vegetation density of habitats are known to influence birdsong evolution due to, respectively, imposing constraints on sound production (Suthers et al. 1999) and affecting sound transmission (Morton 1975; Boncoraglio and Saino 2007). In our data, species with larger body mass sung with slower syllable rate, in accordance with the increased physical inertia of heavier beaks and vocal tracts (Suthers 1994; Podos 2001), and had longer songs, perhaps due to the larger ventilatory capacity of larger birds. Species living in open habitats sung more rattles and trills than species in more densely vegetated habitats, which is consistent with the prediction that forest species should use pure-tone sounds with slow frequency modulation to resist signal degradation by sound refraction on vegetation (Morton 1975; Boncoraglio and Saino 2007).

In conclusion, our study shows that island passerines produce less aggressive elements in songs than mainland species, which is consistent with the hypothesis of reduced territoriality and conspecific aggression on islands (Stamps and Buechner 1985). We did not corroborate predictions that song complexity should decrease, which were expected as a result of reduced mate choice on islands. This parallels the inability of demonstrating relationships between song complexity and strength of sexual selection across passerines (Garamszegi and Møller 2004), and may be due to mate choice targeting multiple song traits that trade-off with song complexity rather than reflecting a real lack of difference in the importance of mate choice on islands compared with mainland areas. In addition, we found an interesting negative effect of the number of closely related sympatric species on song complexity, on islands and continents alike. This result, across a large sample of passerines, concurs with a previous study on suboscines (Seddon 2005) in showing that discrimination against closely related species can be a key factor explaining birdsong evolution.

## Figures and Tables

**Figure A2:**
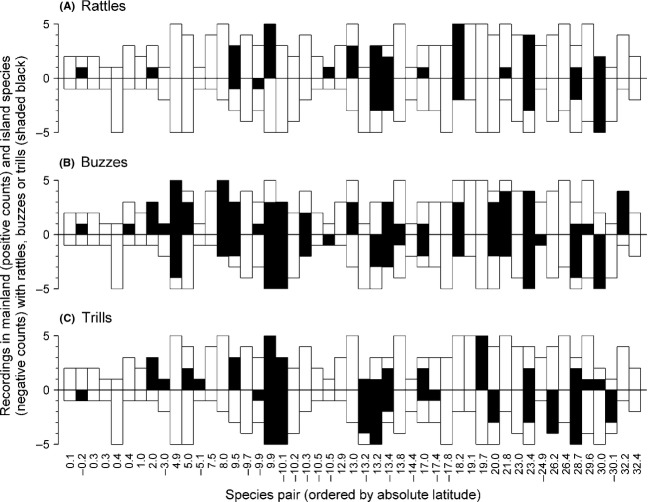
Number of song recordings with (A) rattles, (B) buzzes, and (C) trills in paired mainland and island species (black bars), also showing the total number of recordings per species (black plus white bars). Mean latitude for each species pair is indicated below the horizontal axis (southern latitudes as negative values).

## Appendix

**Table A1:** Species pairs used in this study, ordered by absolute value of latitude of the insular species

Insular species	Latitude	Paired mainland species	Latitude
*Prinia molleri*	0.25	*Prinia bairdii*	0.35
*Zosterops (Speirops) lugubris*	0.25	*Zosterops senegalensis*	0.55
*Serinus rufobrunneus*	0.25	*Serinus striolatus*	−0.67
*Certhidea olivacea*	−0.60	*Tiaris obscura*	10.37
*Myiarchus magnirostris*	−0.60	*Myiarchus tyrannulus*	10.50
*Nesomimus (Mimus) parvulus*	−0.95	*Mimus gilvus*	20.71
*Nesomimus (Mimus) macdonaldi*	−1.45	*Mimus polyglottos*	20.49
*Horizorhinus (Sylvia) dhorni*	1.60	*Pseudoalcippe (Sylvia) abyssinica*	0.35
*Ploceus princeps*	1.60	*Ploceus cucullatus*	2.3
*Copsychus sechellarum*	−4.46	*Copsychus saularis*	4.72
*Pinaroloxias inornata*	5.55	*Tiaris obscura*	10.37
*Rhipidura kubaryi*	6.84	*Rhipidura rufifrons*	−27.22
*Myiagra (oceanica) pluto*	6.84	*Myiagra inquieta*	−35.67
*Colluricincla tenebrosa*	7.28	*Colluricincla megarhyncha*	−28.28
*Myiagra (oceanica) oceanica*	7.43	*Myiagra alecto*	−17.58
*Copsychus cebuensis*	10.30	*Copsychus malabaricus*	4.72
*Turdus bewsheri*	−12.22	*Turdus libonyanus*	−8.82
*Catharopeza bishopi*	13.25	*Setophaga ruticilla*	39.05
*Rhipidura nebulosa*	−13.60	*Rhipidura brachyrhyncha*	−5.70
*Icterus laudabilis*	13.85	*Icterus cayanensis*	−13.32
*Cinclocerthia gutturalis*	13.85	*Melanoptila glabirostris*	20.10
*Myzomela cardinalis*	−14.30	*Myzomela sanguinolenta*	−20.41
*Turdus (Cichlherminia) lherminieri*	15.45	*Turdus plebejus*	10.31
*Saltator albicollis*	15.45	*Saltator striatipectus*	10.58
*Loxigilla portoricensis*	18.20	*Tiaris olivaceus*	8.25
*Icterus portoricensis*	18.20	*Icterus auricapillus*	9.36
*Icterus leucopteryx*	18.20	*Icterus auratus*	20.10
*Vireo latimeri*	18.20	*Vireo pallens*	21.18
*Agelaius xanthomus*	18.20	*Agelaius phoeniceus*	25.36
*Myiarchus antillarum*	18.20	*Myiarchus crinitus*	27.74
*Pachycephala jacquinoti*	−18.60	*Pachycephala pectoralis*	−16.91
*Myiagra vanikorensis*	−19.05	*Myiagra rubecula*	13.09
*Myadestes obscurus*	19.50	*Myadestes townsendi*	39.68
*Acrocephalus rodericanus*	−19.70	*Acrocephalus gracilirostris*	−0.45
*Zosterops chloronothos*	−20.25	*Zosterops senegalensis*	0.55
*Vireo bairdi*	20.40	*Vireo griseus*	19.63
*Toxostoma guttatum*	20.40	*Toxostoma longirostre*	26.42
*Troglodytes (aedon) beani*	20.40	*Troglodytes aedon*	37.09
*Terpsiphone bourbonnensis*	−21.14	*Terpsiphone viridis*	0.59
*Coracina newtoni*	−21.14	*Coracina tenuirostris*	−5.2
*Saxicola tectes*	−21.14	*Saxicola torquatus*	−5.67
*Hypsipetes borbonicus*	−21.14	*Hypsipetes leucocephalus*	21.84
*Zosterops olivaceus*	−21.14	*Zosterops lateralis*	−39.04
*Pomarea dimidiata*	−21.23	*Monarcha melanopsis*	−28.63
*Myadestes myadestinus*	22.50	*Myadestes occidentalis*	13.83
*Geothlypis rostrata*	25.05	*Geothlypis trichas*	27.66
*Tachycineta cyaneoviridis*	26.45	*Tachycineta thalassina*	37.97
*Phylloscopus canariensis*	28.25	*Phylloscopus ibericus*	36.55
*Sylvia conspiciliata orbitalis*	28.42	*Sylvia deserticola*	31.61

**Table A2:** Song traits relative to number of sympatric species, latitude, body mass, and vegetation density of habitats. Results of full and reduced GLMMs paired by species are presented

	Rattles		Buzzes*	Trills*	Song duration*	Frequency bandwidth		Syllable rate*	Syllable diversity*
							
	Full model	Final model	Full model	Full model	Full model	Full model	Final model	Full model	Full model
Same-family sympatric species	= 0.45 (0.502)		= 0.72 (0.395)	= 0.45 (0.502)	*F*_1,38_ = 0.03 (0.859)	*F*_1,38_ = 8.18 (**0.007**)	*F*_1,48_ = 7.61 (**0.008**)	*F*_1,38_ = 0.87 (0.358)	*F*_1,38_ = 1.74 (0.915)
Latitude	= 2.27 (0.132)	= 6.94 (**0.009**)	= 0.05 (0.829)	= 0.13 (0.716)	*F*_1,38_ = 0.02 (0.890)	*F*_1,38_ = 1.08 (0.305)		*F*_1,38_ = 0.01 (0.920)	*F*_1,38_ = 0.05 (0.831)
Mass	= 1.37 (0.243)		= 0.05 (0.825)	= 0.253 (0.615)	*F*_1,38_ = 10.3 (**0.003**)	*F*_1,38_ = 1.55 (0.220)		*F*_1,38_ = 11.4 (**0.002**)	*F*_1,38_ = 0.01 (0.946)
Vegetation density	= 7.14 (**0.028**)	= 11.2 (**0.004**)	= 1.19 (0.551)	= 6.40 (**0.041**)	*F*_1,38_ = 1.44 (0.250)	*F*_1,38_ = 0.51 (0.607)		*F*_1,38_ = 1.56 (0.223)	*F*_1,38_ = 0.07 (0.930)

Indicated are χ² or *F* statistics for each factor or covariate and *P*-values (significant values in bold).

* No significant factors or covariates after model selection for these song traits.
